# Immunogenicity and safety of trivalent inactivated influenza vaccine in adults living with HIV compared with HIV-uninfected adults: a study from China

**DOI:** 10.3389/fimmu.2026.1945123

**Published:** 2026-10-16

**Authors:** Jinfeng Su, Kai Gao, Shasha Han, Xuewen Tang, Xue Guo, Hui Liang, Xiaoqin Liu, Xiaoteng Xu, Jun Yan, Ye Qiu, Ye Yao, Dingyan Yan, Baiqing Zhao, Kexing Zhou, Chenlu Xu, Xiaohua Qi, Yehong Wu, Jianhua Yu

**Affiliations:** 1 Peking University, Beijing, China; 2 Department of Marketing and Product Planning, China National Biotec Group Company Limited, Beijing, China; 3 Medical Affairs Department, China National Biotec Group Company Limited, Beijing, China; 4 Department of Immunization Program, Zhejiang Provincial Center for Disease Control and Prevention, Hangzhou, China; 5 Medical Affairs Department, Changchun Institute of Biological Products Co., Ltd, Changchun, China; 6 Research and Development Department, Changchun Institute of Biological Products Co., Ltd, Changchun, China; 7 State Key Laboratory of Novel Vaccines for Emerging Infectious Diseases, Beijing, China; 8 Second Department of Infectious Diseases, Hangzhou Xixi Hospital of Hangzhou Medical College, Hangzhou, China; 9 Second Department of Hepatology, Hangzhou Xixi Hospital of Hangzhou Medical College, Hangzhou, China; 10 Emergency Department, Hangzhou Xixi Hospital of Hangzhou Medical College, Hangzhou, China; 11 Department of Infection Prevention and Control, Hangzhou Xixi Hospital of Hangzhou Medical College, Hangzhou, China; 12 School of Public Health, Fudan University, Shanghai, China; 13 Zhejiang Key Lab of Vaccine, Infectious Disease Prevention and Control, Hangzhou, China; 14 Department of Infectious Diseases, Hangzhou Xixi Hospital of Hangzhou Medical College, Hangzhou, China

**Keywords:** HIV infection, immunogenicity, safety, seroprotection, trivalent inactivated influenza vaccine

## Abstract

**Objective:**

To evaluate the immunogenicity and safety of trivalent inactivated influenza vaccine (IIV3) in adults aged ≥18 years living with HIV and to provide evidence supporting influenza vaccination in this population in China.

**Methods:**

This single-center, open-label comparative analysis was derived from a larger three-arm clinical study. For the present comparative analysis, 472 vaccinated participants were planned, including 236 adults living with HIV (Group 1) and 236 age- and sex-comparable HIV-uninfected adults (Group 2). Participants in both groups received one dose of IIV3 (0.5 mL) on Day 0. Blood samples were collected before and 28 days after vaccination for hemagglutination inhibition (HI) antibody testing against A(H1N1), A(H3N2), and B/Victoria strains. Immunogenicity endpoints included seroconversion rate, seroprotection rate, geometric mean titer (GMT), and geometric mean increase (GMI). Safety was assessed by monitoring solicited and unsolicited adverse events (AEs) and serious adverse events (SAEs) during 6 months of follow-up. An unvaccinated cohort of adults living with HIV from the original trial was included only for an exploratory, descriptive analysis of influenza-like illness (ILI), without assessment of vaccine efficacy.

**Results:**

A total of 489 participants were enrolled, including 237 in Group 1 and 252 in Group 2. At 28 days post-vaccination, seroconversion rates did not differ significantly between Group 1 and Group 2 for A(H1N1) (91.00% vs. 86.69%, *P* > 0.05), but significantly lower for A(H3N2) (49.50% vs. 89.52%) and B/Victoria (40.00% vs. 64.52%) (both *P* < 0.001). Seroprotection rates were also lower in Group 1 than in Group 2 for A(H1N1), A(H3N2), and B/Victoria (94.00% vs. 97.98%, 63.50% vs. 97.18%, and 40.50% vs. 68.55%, respectively). Post-vaccination GMTs were significantly lower in Group 1 for all three strains (all *P* < 0.001). GMI was higher for A(H1N1) but lower for A(H3N2) and B/Victoria in Group 1 than in Group 2 (all *P* < 0.001). Multivariable analyses showed that HIV infection remained independently associated with several measures of vaccine-induced immune responses after adjustment for demographic and clinical factors, particularly for A(H3N2). IIV3 was generally safe and well tolerated. Most AEs were mild in severity, and no Grade 4 adverse reactions or vaccine-related SAEs were reported. During the period from 29 days to 6 months after vaccination, few influenza-like illness events were reported among vaccinated and unvaccinated participants living with HIV; these findings were descriptive and were not used to assess vaccine efficacy against influenza.

**Conclusion:**

Adults living with HIV developed substantial antibody responses following IIV3 vaccination, although the magnitude of response varied across influenza strains. A(H1N1) seroconversion was high in adults living with HIV, whereas responses to A(H3N2) and B/Victoria were generally lower than those in HIV-uninfected adults. HIV infection remained associated with reduced vaccine-induced immune responses for several strain-specific outcomes after multivariable adjustment, with associations particularly evident for A(H3N2). IIV3 was safe and well tolerated, supporting current recommendations for influenza vaccination in adults living with HIV.

**Clinical trial registration:**

https://www.chictr.org.cn/showprojEN.html?proj=255613, identifier ChiCTR2500112191.

## Highlights

Adults living with HIV showed high A(H1N1) seroconversion following IIV3 vaccination. Responses to A(H3N2) and B/Victoria were reduced compared with HIV-uninfected adults. HIV infection was associated with reduced vaccine-induced immune responses after multivariable adjustment. Baseline HI antibody titer was consistently associated with post-vaccination responses. IIV3 was well tolerated, with no vaccine-related serious adverse events.

## Introduction

Seasonal influenza remains a major global public health concern, causing substantial morbidity and mortality worldwide (1, 2). According to the World Health Organization (WHO), influenza causes approximately 1 billion infections annually, including 3–5 million severe cases and 290, 000–650, 000 influenza-associated respiratory deaths (2). Influenza-related morbidity and mortality are particularly high among vulnerable populations, including older adults, children, pregnant women, individuals with chronic medical conditions, and immunocompromised persons (2, 3).

Despite widespread use of antiretroviral therapy (ART), people living with HIV (PLWH) remain particularly susceptible to severe influenza outcomes because of persistent immune dysregulation and a higher prevalence of comorbidities (4, 5). Consequently, annual influenza vaccination is recommended for PLWH by both WHO and the U.S. Centers for Disease Control and Prevention (CDC) (3, 6).

Although previous studies have demonstrated that PLWH can mount antibody responses following influenza vaccination, vaccine-induced immunity is often lower than that observed in HIV-uninfected adults and may be influenced by factors such as age, CD4+ T-cell count, HIV viral load, ART status, and baseline antibody levels (4, 5, 7, 8) In addition, immune responses may vary across influenza virus strains, and evidence regarding influenza vaccine immunogenicity and safety among adults living with HIV in China remains scarce.

Trivalent inactivated influenza vaccine (IIV3) has been widely used for seasonal influenza prevention and typically contains antigens against two influenza A subtypes, A(H1N1) and A(H3N2), and one influenza B lineage. The vaccine evaluated in this study was a licensed split-virion IIV3 manufactured by Changchun Institute of Biological Products Co., Ltd. in China and indicated for the prevention of seasonal influenza in individuals aged ≥3 years. Immunogenicity is commonly evaluated using hemagglutination inhibition (HI) antibody responses, including seroconversion rates, seroprotection rates, geometric mean titers (GMTs), and geometric mean increases (GMIs). However, data regarding the immunogenicity and safety of IIV3 among Chinese adults living with HIV remain limited.

Therefore, this study aimed to evaluate the immunogenicity and safety of IIV3 in adults living with HIV in China and to compare vaccine-induced immune responses with those in HIV-uninfected adults. The findings may provide additional evidence to support influenza vaccination strategies for immunocompromised populations in China.

## Methods

### Study design and participants

This study was a single-center, open-label comparative analysis derived from a larger three-arm clinical study conducted in Hangzhou, Zhejiang Province, China, in which participants living with HIV were randomized to vaccination or no vaccination. Participant recruitment was conducted at Hangzhou Xixi Hospital of Hangzhou Medical College and three community health service centers in Xihu District, Hangzhou. Participants living with HIV and HIV-uninfected participants were enrolled concurrently during the same study period. The present analysis evaluated the immunogenicity and safety of IIV3 in adults living with HIV and compared vaccine-induced immune responses with those in an age- and sex-comparable group of HIV-uninfected adults.

For the present comparative analysis, 472 vaccinated participants were planned, including 236 adults living with HIV (Group 1) and 236 age- and sex-comparable HIV-uninfected adults (Group 2). Participants living with HIV were stratified by age (18–49 years and ≥50 years) and recruited primarily from the study hospital, whereas HIV-uninfected adults were recruited from both hospital- and community-based settings. Group 2 was selected to be age- and sex-comparable to Group 1 without individual matching. All participants included in the present analysis received a single 0.5 mL dose of IIV3 administered intramuscularly into the deltoid muscle on Day 0.

Blood samples were collected before vaccination (Day 0) and 28 days after vaccination to assess HI antibody responses. Participants were provided with diary cards to record adverse events (AEs) following vaccination. Solicited and unsolicited AEs were monitored after vaccination, and serious adverse events (SAEs) were collected throughout the 6-month follow-up period.

The original trial also included an unvaccinated cohort of adults living with HIV. Data from this cohort were used only for an exploratory, descriptive analysis of influenza-like illness (ILI) during follow-up. Because ILI was not virologically confirmed, this analysis was considered hypothesis-generating and was not intended to assess vaccine efficacy against influenza. At the time of study conduct, seasonal influenza vaccination in China was voluntary and outside the National Immunization Program. Chinese technical guidelines recommended annual influenza vaccination for all individuals aged ≥6 months without contraindications, with priority given to key and high-risk populations (9). The inclusion of the unvaccinated group of adults living with HIV was considered in this policy context, and randomization concerned receipt of the study influenza vaccine only without affecting routine HIV care or antiretroviral therapy. The parent study protocol was approved by the relevant ethics committees, and all participants provided written informed consent.

Participants included in the present analysis were adults aged ≥18 years who had not received any influenza vaccine within the year preceding enrollment and had a body temperature <37.3 °C at enrollment. Female participants of childbearing potential were required to use effective contraception and have no pregnancy plans during the study period.

Key exclusion criteria included a history of severe allergic reactions to influenza vaccines or vaccine components, pregnancy or breastfeeding, use of immunosuppressive agents within 1 month before enrollment, receipt of blood products or immunoglobulins within 3 months before enrollment, acute febrile illness, Guillain–Barré syndrome, coagulation disorders contraindicating intramuscular injection, or any condition that, in the investigator’s judgment, could interfere with study assessments.

### Study endpoints

The primary endpoint was the seroconversion rate against influenza A(H1N1), A(H3N2), and B/Victoria strains in Group 1 at 28 days after vaccination. Secondary endpoints included comparisons of seroconversion rates, seroprotection rates, GMTs, and GMIs between Group 1 and Group 2. Exploratory endpoints included evaluation of demographic and clinical factors associated with vaccine-induced immune responses. Additional analyses among participants living with HIV evaluated recent CD4+ T-cell count, time since HIV diagnosis, and duration of ART. Safety endpoints included AEs and SAEs following vaccination. ILI occurrence was also summarized descriptively as an exploratory, hypothesis-generating observation among vaccinated and unvaccinated participants living with HIV. ILI was defined as fever (≥38.0 °C) accompanied by cough or sore throat. ILI was identified based on clinical symptoms without virological confirmation and was therefore not used to assess vaccine efficacy against influenza.

### Sample size calculation and randomization

This analysis was derived from a larger three-arm, single-center, open-label clinical study. The original sample size was calculated assuming seroconversion rates of 40% and 23% in vaccinated and unvaccinated adults living with HIV, respectively. With a two-sided α of 0.05, 95% power, and an anticipated dropout rate of 18%, 236 participants were required per group.

In the original trial, participants living with HIV were stratified by age (18–49 years and ≥50 years) and randomly assigned to either the vaccinated or unvaccinated group using a stratified randomization sequence generated with SAS version 9.4 (SAS Institute, Cary, NC, USA). HIV-uninfected adults were enrolled separately as an age- and sex-comparable comparison group. For the present analysis, only vaccinated adults living with HIV and vaccinated HIV-uninfected adults were included.

### Study vaccine

The IIV3 (Shipulier^®^) was manufactured by Changchun Institute of Biological Products Co., Ltd. (Changchun, China). Each dose (0.5 mL per vial, batch number: TA20240725, expiration date: July 17, 2025) contained 15 μg of hemagglutinin antigen from each influenza virus strain included in the vaccine formulation. The IIV3 formulation corresponded to the WHO-recommended egg-based trivalent influenza vaccine composition for the 2024–2025 Northern Hemisphere season (10).

### Immunogenicity assessment

Immunogenicity was evaluated using the hemagglutination inhibition (HI) assay performed at the Bioanalytical Center of Chongqing Meilaide Biopharmaceutical Co., Ltd. Serum HI antibody titers against influenza A(H1N1) (A/Victoria/4897/2022), A(H3N2) (A/Thailand/8/2022), and B/Victoria (B/Austria/1359417/2021) vaccine strains were measured before vaccination (Day 0) and 28 days after vaccination.

Immunogenicity endpoints included seroconversion rate, seroprotection rate, GMTs, and GMIs. Seroconversion was defined as a pre-vaccination HI antibody titer <1:10 and a post-vaccination titer ≥1:40, or a pre-vaccination titer ≥1:10 with at least a four-fold increase after vaccination. Seroconversion rate was defined as the proportion of participants who achieved seroconversion. Seroprotection was defined as an HI antibody titer ≥1:40, and the seroprotection rate was defined as the proportion of participants achieving this threshold at the specified time point. GMTs were calculated for each influenza strain before and 28 days after vaccination. GMI was calculated as the geometric mean of the individual post-vaccination-to-pre-vaccination HI antibody titer ratios.

### Safety assessment

Safety assessments included all solicited and unsolicited AEs, regardless of their relationship to vaccination. Participants were observed for 30 minutes after vaccination and were provided with diary cards to record AEs occurring within 28 days after vaccination. Solicited local (injection-site pain, induration, swelling, rash, erythema, and pruritus) and systemic (fever, cough, headache, myalgia, arthralgia, fatigue, nausea, vomiting, and acute allergic reactions) AEs were collected during the first 7 days following vaccination. Any medical occurrence other than the predefined solicited events was recorded as an unsolicited AE through Day 28. SAEs were monitored for 6 months through active surveillance, consisting of scheduled on-site or telephone follow-up every 7 days, supplemented by spontaneous reporting from participants.

Safety analyses compared the incidence, severity, and causality of AEs, as well as the incidence of SAEs, between Group 1 and Group 2. All reported AEs were graded for severity and assessed for causality by trained investigators according to the Guiding Principles for Grading Adverse Reactions in Clinical Trials of Preventive Vaccines (NMPA No. 102, 2019) (11).

For participants living with HIV, planned hospitalizations or hospitalizations for routine clinical management that were scheduled before enrollment and did not reflect a worsening medical condition were not classified as SAEs, in accordance with the study protocol. All other events meeting the protocol-defined SAE criteria were reported and evaluated by the investigators.

### Statistical analysis

Statistical analyses were performed using SAS version 9.4 (SAS Institute, Cary, NC, USA). GMTs, GMIs, and corresponding 95% confidence intervals (CIs) were calculated from log_10_-transformed HI antibody titers and back-transformed for presentation. Seroconversion and seroprotection rates were calculated with corresponding 95% CIs. Comparisons between groups were performed using the chi-square test, Fisher’s exact test, or t-test, as appropriate. Multivariable logistic regression analyses were used to identify factors associated with seroconversion and seroprotection, whereas multivariable linear regression analyses were performed using individual-level log_2_-transformed post-vaccination HI antibody titers and log_2_-transformed antibody fold increases as continuous outcomes. Separate models were fitted for each influenza strain. Among vaccinated participants living with HIV and HIV-uninfected participants, models included age (18–49 vs. ≥50 years), sex, HIV infection status, comorbidity, and baseline log_2_ HI antibody titer. In exploratory analyses restricted to vaccinated participants living with HIV, models included age, sex, comorbidity, baseline log_2_ HI antibody titer, recent CD4+ T-cell count (<500 vs. ≥500 cells/μL), time since HIV diagnosis (<5 vs. ≥5 years), and duration of ART (<2 vs. ≥2 years). Baseline log_2_ HI antibody titer was modeled as a continuous variable.

As a complementary analysis of the overall humoral response across the three vaccine strains, a global three-strain response score was constructed using an approach adapted from Tawfik et al. (12). HI titers for A(H1N1), A(H3N2), and B/Victoria were transformed into ordinal scores, combined with a seroprotection-weighting component, and normalized using an ordered quantile (ORQ) transformation. A repeated-measures linear mixed-effects model was fitted with the global response score as the outcome, Group, Time, and Group × Time as fixed effects, and participant as a random intercept. At Day 28, the distribution of the number of strains achieving seroprotection (HI ≥1:40; 0–3 strains) was compared between groups using the Pearson chi-square test, and the proportion achieving seroprotection against all three strains was compared using the Pearson chi-square test with Yates’ continuity correction. The complementary global-response analyses were performed using Python version 3.12.

All statistical tests were two-sided, and *P* < 0.05 was considered statistically significant.

### Analysis sets

The full analysis set (FAS) included all vaccinated participants with evaluable pre- and post-vaccination HI antibody results. The per-protocol set (PPS) included participants who completed the study without major protocol deviations and had evaluable immunogenicity data at both study visits. Safety analyses were performed using the safety set (SS), which included all vaccinated participants.

## Results

### Study participants

Between December 3, 2024 and February 27, 2025, 489 participants were enrolled, including 237 adults living with HIV (Group 1) and 252 HIV-uninfected adults (Group 2). A total of 237 and 251 participants were included in the SS, 223 and 250 in the FAS, and 200 and 248 in the PPS in Groups 1 and 2, respectively. No statistically significant between-group differences were observed in age, sex, ethnicity, or the overall prevalence of comorbidities (**Table 1**). Any comorbidity was reported in 26.00% of Group 1 and 24.19% of Group 2 (*P* = 0.663). Hypertension and diabetes mellitus were the most common comorbidities in both groups. Detailed baseline comorbidity profiles and between-group comparisons are presented in **Supplementary Table 1**. The participant flow is shown in **Figure 1**.

**Table 1:** Baseline characteristics of participants in the PPS.

Characteristic	Group 1 (n=200)	Group 2 (n=248)	*P* value
Age, years, mean ± SD	45.60 ± 12.13	45.62 ± 13.31	0.986
Male sex, n (%)	196 (98.00)	244 (98.39)	1.000
Ethnicity, Han, n (%)	198 (99.00)	247 (99.60)	0.589
Comorbidity, n (%)	52 (26.00)	60 (24.19)	0.663

PPS, per-protocol set; SD, standard deviation.

Data are presented as mean ± SD or n (%), as appropriate.

**Figure 1:**
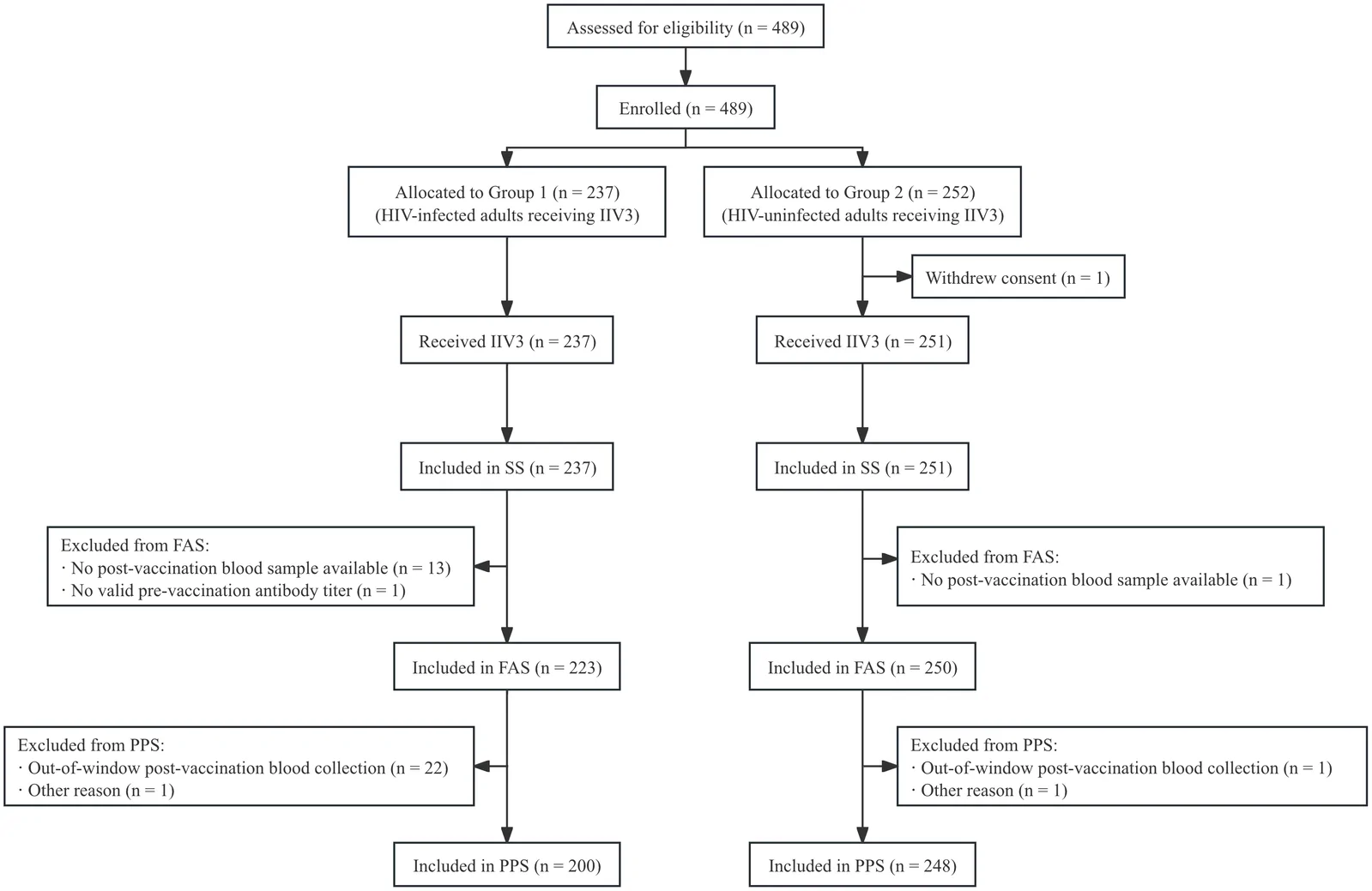
Participant flow diagram. IIV3, trivalent inactivated influenza vaccine; SS, safety set; FAS, full analysis set; PPS, per-protocol set.

### Immunogenicity

At 28 days after vaccination, Group 1 achieved high seroconversion and seroprotection rates against A(H1N1), with no statistically significant difference in seroconversion rates between Group 1 and Group 2 (91.00% vs. 86.69%, *P* = 0.154). In contrast, seroconversion rates against A(H3N2) and B/Victoria were significantly lower in Group 1 than in Group 2 (49.50% vs. 89.52% and 40.00% vs. 64.52%, respectively; both *P* < 0.001). Similar patterns were observed for seroprotection rates. Although seroprotection against A(H1N1) remained high among adults living with HIV (94.00%), it was significantly lower than that observed among HIV-uninfected adults (97.98%, *P* = 0.028) (**Table 2**).

**Table 2:** Immunogenicity of IIV3 at 28 days after vaccination in the PPS.

Outcome	Group 1 (n=200)	Group 2 (n=248)	*P* value
Seroconversion rate, n/N (%) [95% CI]			
A(H1N1)	182/200 (91.00) [86.15–94.58]	215/248 (86.69) [82.47–90.92]	0.154
A(H3N2)	99/200 (49.50) [42.57–56.43]	222/248 (89.52) [85.70–93.33]	<0.001
B/Victoria	80/200 (40.00) [33.21–46.79]	160/248 (64.52) [58.56–70.47]	<0.001
Seroprotection rate, n/N (%) [95% CI]			
A(H1N1)	188/200 (94.00) [89.75–96.86]	243/248 (97.98) [96.23–99.73]	0.028
A(H3N2)	127/200 (63.50) [56.42–70.18]	241/248 (97.18) [95.12–99.24]	<0.001
B/Victoria	81/200 (40.50) [33.63–47.65]	170/248 (68.55) [62.77–74.33]	<0.001
Pre-vaccination GMT (95% CI)			
A(H1N1)	7.45 (6.70–8.28)	22.30 (19.00–26.18)	<0.001
A(H3N2)	16.02 (13.64–18.82)	23.92 (20.75–27.57)	<0.001
B/Victoria	5.36 (5.11–5.62)	6.34 (5.82–6.90)	<0.001
Post-vaccination GMT (95% CI)			
A(H1N1)	240.84 (197.22–294.10)	438.85 (378.41–508.93)	<0.001
A(H3N2)	65.89 (50.47–86.02)	330.91 (282.69–387.37)	<0.001
B/Victoria	25.85 (20.52–32.56)	60.16 (49.80–72.66)	<0.001
GMI (95% CI)			
A(H1N1)	32.33 (26.55–39.39)	19.68 (16.21–23.88)	<0.001
A(H3N2)	4.11 (3.27–5.18)	13.84 (11.74–16.30)	<0.001
B/Victoria	4.82 (3.85–6.04)	9.49 (7.88–11.42)	<0.001

IIV3, trivalent inactivated influenza vaccine; PPS, per-protocol set; CI, confidence interval; GMT, geometric mean titer; GMI, geometric mean increase.

Seroconversion was defined as a pre-vaccination HI titer <1:10 and a post-vaccination titer ≥1:40, or at least a four-fold increase in HI titer after vaccination.

Pre-vaccination and post-vaccination GMTs were significantly lower in Group 1 than in Group 2 for all three influenza strains (all *P* < 0.001). Although the GMI against A(H1N1) was significantly higher in Group 1 than in Group 2 (32.33 vs. 19.68, *P* < 0.001), GMIs against A(H3N2) and B/Victoria were significantly lower in Group 1 (both *P* < 0.001) (**Table 2**).

Subgroup analyses stratified by baseline HI antibody titers (<1:10 vs. ≥1:10 and <1:40 vs. ≥1:40) yielded findings generally consistent with the overall analysis. For A(H3N2), adults living with HIV consistently demonstrated lower seroconversion rates, seroprotection rates, GMTs, and GMIs than HIV-uninfected adults across all baseline antibody strata, suggesting that lower immune responses among participants living with HIV persisted across baseline antibody strata. In contrast, most between-group differences in A(H1N1) immune responses were not statistically significant after stratification. For B/Victoria, between-group differences were primarily observed among participants with lower baseline antibody titers, whereas most between-group differences were not statistically significant among participants with higher baseline antibody levels. Similar findings were observed using both baseline HI antibody titer cutoffs (<1:10 vs. ≥1:10 and <1:40 vs. ≥1:40) (**Supplementary Tables 2**, **3**).

### Global three-strain humoral response

As a complementary analysis integrating HI responses across all three vaccine strains, the global three-strain response score increased from Day 0 to Day 28 in both groups (**Supplementary Table 4**). The mean ± SD score increased from 1.14 ± 1.41 to 4.77 ± 1.45 in Group 1 and from 2.39 ± 1.35 to 6.27 ± 1.50 in Group 2. In the repeated-measures mixed-effects model, the Time effect was significant (estimate, 3.879; 95% CI, 3.674–4.084; *P* < 0.001), whereas the Group × Time interaction was not statistically significant (estimate, −0.256; 95% CI, −0.563 to 0.052; *P* = 0.103). The overall Group effect was significant (estimate, −1.249; 95% CI, −1.515 to −0.983; *P* < 0.001), with lower global response scores in Group 1 across the two time points; however, the groups already differed at Day 0. A model-based between-group contrast at Day 28 showed that the estimated global three-strain response score remained significantly lower among adults living with HIV than among HIV-uninfected adults (estimated difference, −1.505; 95% CI, −1.770 to −1.239; *P* < 0.001; **Supplementary Table 4**). The distributions of individual global three-strain response scores at Day 0 and Day 28 in both groups are shown in **Supplementary Figure 1**.

At Day 28, the distribution of the number of strains achieving seroprotection (HI ≥1:40) differed between groups (*P* < 0.001; **Supplementary Table 5**). Seroprotection against all three strains was achieved by 44/200 (22.00%) participants in Group 1 and 164/248 (66.13%) participants in Group 2 (*P* < 0.001).

### Factors associated with vaccine-induced immune responses

To identify factors associated with vaccine-induced immune responses, multivariable analyses were performed. Multivariable analyses showed that HIV infection was independently associated with several vaccine-induced immune response outcomes across the three strains (**Table 3**). For A(H3N2) and B/Victoria, HIV infection was associated with significantly lower odds of seroconversion and seroprotection, as well as lower post-vaccination HI antibody titers and antibody increases. For A(H1N1), HIV infection was associated with lower odds of seroconversion and lower post-vaccination HI antibody titers and antibody increases, but not with seroprotection. Baseline log_2_ HI antibody titer was positively associated with seroprotection and post-vaccination antibody titers across most analyses, whereas higher baseline antibody levels were associated with smaller antibody increases after vaccination. Older age was associated with reduced immune responses against A(H3N2) but higher responses against B/Victoria, while male sex and comorbidity showed limited or strain-specific associations (**Table 3**).

**Table 3:** Multivariable analyses of factors associated with vaccine-induced HI antibody responses among IIV3 recipients in the PPS.

Variable	A(H1N1)		A(H3N2)		B/Victoria	
	Estimate (95% CI)	*P* value	Estimate (95% CI)	*P* value	Estimate (95% CI)	*P* value
Seroconversion, aOR (95% CI)						
Age ≥50 years	1.89 (0.87−4.11)	0.107	0.43 (0.26−0.72)	0.001	2.05 (1.34−3.14)	<0.001
Male sex	8.86 (1.60−49.00)	0.012	0.47 (0.08−2.86)	0.412	0.92 (0.20−4.19)	0.915
HIV infection	0.44 (0.20−0.99)	0.046	0.10 (0.06−0.17)	<0.001	0.35 (0.24−0.53)	<0.001
Comorbidity	0.45 (0.20−1.01)	0.053	1.16 (0.65−2.06)	0.612	1.01 (0.62−1.67)	0.961
Baseline log_2_ HI titer	0.53 (0.43−0.65)	<0.001	0.94 (0.82−1.09)	0.427	0.94 (0.74−1.20)	0.617
Seroprotection, aOR (95% CI)						
Age ≥50 years	2.44 (0.79−7.48)	0.120	0.23 (0.11−0.45)	<0.001	1.94 (1.24−3.04)	0.004
Male sex	11.93 (0.96−148.80)	0.054	0.20 (0.02−2.36)	0.203	1.38 (0.26−7.28)	0.706
HIV infection	0.93 (0.30−2.89)	0.896	0.05 (0.02−0.11)	<0.001	0.34 (0.22−0.51)	<0.001
Comorbidity	0.30 (0.10−0.93)	0.037	0.98 (0.48−2.00)	0.945	1.11 (0.66−1.89)	0.689
Baseline log_2_ HI titer	4.33 (1.33−14.06)	0.015	2.22 (1.69−2.93)	<0.001	11.83 (2.86−48.88)	<0.001
GMT, β (95% CI)						
Age ≥50 years	-0.09 (-0.47−0.29)	0.635	-1.15 (-1.55− -0.75)	<0.001	0.62 (0.17−1.07)	0.007
Male sex	1.00 (-0.30−2.30)	0.130	-0.73 (-2.14−0.67)	0.306	0.78 (-0.79−2.35)	0.328
HIV infection	-0.46 (-0.84−-0.07)	0.020	-1.97 (-2.34 −-1.59)	<0.001	-1.06 (-1.48− -0.64)	<0.001
Comorbidity	-0.46 (-0.89−-0.03)	0.034	0.33 (-0.14−0.79)	0.168	-0.02 (-0.54−0.50)	0.946
Baseline log_2_ HI titer	0.27 (0.15−0.38)	<0.001	0.60 (0.49−0.71)	<0.001	0.65 (0.39−0.91)	<0.001
GMI, β (95% CI)						
Age ≥50 years	-0.09 (-0.47−0.29)	0.635	-1.15 (-1.55−-0.75)	<0.001	0.62 (0.17− 1.07)	0.007
Male sex	1.00 (-0.30−2.30)	0.130	-0.73 (-2.14−0.67)	0.306	0.78 (-0.79−2.35)	0.328
HIV infection	-0.46 (-0.84−-0.07)	0.020	-1.97 (-2.34−-1.59)	<0.001	-1.06 (-1.48−-0.64)	<0.001
Comorbidity	-0.46 (-0.89−-0.03)	0.034	0.33 (-0.14− 0.79)	0.168	-0.02 (-0.54−0.50)	0.946
Baseline log_2_ HI titer	-0.74 (-0.85−-0.62)	<0.001	-0.40 (-0.51− -0.29)	<0.001	-0.35 (-0.61−-0.09)	0.008

HI, hemagglutination inhibition; GMT, geometric mean titer; GMI, geometric mean increase; PPS, per-protocol set; aOR, adjusted odds ratio; β, regression coefficient; CI, confidence interval.

Multivariable logistic regression was used for seroconversion and seroprotection, and multivariable linear regression was used for post-vaccination HI antibody titers and GMI. Models included age, sex, HIV infection status, comorbidity, and baseline log_2_ HI antibody titer. Reference categories were age 18−49 years, female sex, HIV-uninfected status, and no comorbidity. Baseline log_2_ HI antibody titer was modeled as a continuous variable.

Among participants living with HIV, all participants in the PPS were receiving ART. The median recent CD4+ T-cell count was 557 cells/μL (IQR, 394–702), and 199/200 participants had evaluable HIV RNA results, all of whom were virologically suppressed (<200 copies/mL). Additional HIV-related clinical characteristics are presented in **Supplementary Table 6**. Exploratory multivariable analyses demonstrated that recent CD4+ T-cell count, time since HIV diagnosis, and ART duration were not significantly associated with vaccine-induced immune responses after adjustment for other covariates (**Supplementary Table 7**). Baseline log_2_ HI antibody titer remained consistently associated with post-vaccination immune responses, whereas older age was associated with lower immune responses against A(H3N2) and higher immune responses against B/Victoria, with no significant association observed for A(H1N1).

### Safety

No vaccine-related SAEs were reported during the 6-month follow-up period (**Table 4**). No statistically significant between-group differences were observed in the incidences of vaccine-related or overall AEs within 30 minutes or 7 days after vaccination. By Day 28, vaccine-related AEs and overall AEs were reported more frequently in Group 1 than in Group 2 (16.46% vs. 10.36%, *P* = 0.048; 18.57% vs. 11.95%, *P* = 0.042, respectively) (**Table 4**).

**Table 4:** Incidence of adverse events within 30 minutes, 7 days, and 28 days after vaccination in the SS.

AEs	Vaccine-related			Overall		
	Group 1 (n=237)	Group 2 (n=251)	*P* value	Group 1 (n=237)	Group 2 (n=251)	*P* value
30 min, n (%)	7 (2.95)	8 (3.19)	0.881	7 (2.95)	8 (3.19)	0.881
7 days, n (%)	26 (10.97)	19 (7.57)	0.194	28 (11.81)	21 (8.37)	0.205
28 days, n (%)	39 (16.46)	26 (10.36)	0.048	44 (18.57)	30 (11.95)	0.042
SAEs within 6 months, n (%)	0 (0.00)	0 (0.00)	–	0 (0.00)	0 (0.00)	–

SS, safety set; AE, adverse event; SAE, serious adverse event.

Data are presented as n (%). Vaccine-related adverse events were defined as adverse events considered by the investigators to be related to vaccination. *P* values were calculated using the chi-square test or Fisher’s exact test, as appropriate.

Injection-site pain was the most frequently reported solicited local AE in both groups. The incidence of vaccine-related solicited systemic AEs within 7 days after vaccination was significantly higher in Group 1 than in Group 2 (5.49% vs. 1.20%, *P* = 0.008). Among systemic AEs, cough, myalgia, and fatigue were reported more frequently in Group 1. In addition, vaccine-related unsolicited AEs occurred more frequently in Group 1 than in Group 2 (5.91% vs. 0.80%, *P* = 0.002) (**Table 5**).

**Table 5:** Solicited local and systemic adverse events within 7 days after vaccination in the SS.

AEs	Vaccine-related			Overall		
	Group 1 (n=237)	Group 2 (n=251)	*P* value	Group 1 (n=237)	Group 2 (n=251)	*P* value
Local, n (%)	12 (5.06)	16 (6.37)	0.534	12 (5.06)	16 (6.37)	0.534
injection-site pain	12 (5.06)	15 (5.98)	0.659	12 (5.06)	15 (5.98)	0.659
induration	6 (2.53)	3 (1.20)	0.327	6 (2.53)	3 (1.20)	0.327
swelling	6 (2.53)	3 (1.20)	0.327	6 (2.53)	3 (1.20)	0.327
rash	2 (0.84)	0 (0.00)	0.235	2 (0.84)	0 (0.00)	0.235
erythema	4 (1.69)	1 (0.40)	0.204	4 (1.69)	1 (0.40)	0.204
pruritus	3 (1.27)	0 (0.00)	0.114	3 (1.27)	0 (0.00)	0.114
Systemic, n (%)	13 (5.49)	3 (1.20)	0.008	13 (5.49)	6 (2.39)	0.077
fever	2 (0.84)	0 (0.00)	0.235	2 (0.84)	0 (0.00)	0.235
cough	8 (3.38)	1 (0.40)	0.017	8 (3.38)	2 (0.80)	0.057
headache	2 (0.84)	1 (0.40)	0.614	2 (0.84)	1 (0.40)	0.614
myalgia	7 (2.95)	1 (0.40)	0.033	7 (2.95)	2 (0.80)	0.098
arthralgia	1 (0.42)	1 (0.40)	1.000	1 (0.42)	1 (0.40)	1.000
fatigue	7 (2.95)	0 (0.00)	0.006	7 (2.95)	2 (0.80)	0.098
nausea	1 (0.42)	0 (0.00)	0.486	1 (0.42)	0 (0.00)	0.486
vomiting	1 (0.42)	0 (0.00)	0.486	1 (0.42)	0 (0.00)	0.486
acute allergic reactions	1 (0.42)	0 (0.00)	0.486	1 (0.42)	0 (0.00)	0.486
Unsolicited adverse events, n (%)	14 (5.91)	2 (0.80)	0.002	16 (6.75)	2 (0.80)	<0.001

SS, safety set; AE, adverse event.

Data are presented as n (%). Participants may have experienced more than one adverse event. Solicited local adverse events included injection-site pain, induration, swelling, rash, erythema, and pruritus. Solicited systemic adverse events included fever, cough, headache, myalgia, arthralgia, fatigue, nausea, vomiting, and acute allergic reactions. *P* values were calculated using the chi-square test or Fisher’s exact test, as appropriate.

Most vaccine-related adverse reactions were mild to moderate in severity, with Grade 1 events accounting for the majority of reported reactions. Grade ≥3 vaccine-related adverse reactions were uncommon, and no Grade 4 adverse reactions were observed in either group (**Supplementary Table 8**).

### Exploratory analysis of influenza-like illness

During the period from 29 days to 6 months after vaccination, few ILI events were reported, occurring in 0.50% of vaccinated participants living with HIV and 0.81% of unvaccinated participants living with HIV (**Supplementary Table 9**). Given the small number of events and the absence of virological confirmation, these findings are presented descriptively as hypothesis-generating observations and should not be interpreted as evidence of vaccine efficacy against influenza.

## Discussion

Seasonal influenza remains an important cause of morbidity in adults living with HIV, and international guidelines recommend annual influenza vaccination for people living with HIV (6, 13). Previous systematic reviews have shown that influenza vaccines are generally safe and immunogenic in adults living with HIV, although immune responses vary across influenza strains and study populations (5, 14). More recently, a Chinese comparative study reported satisfactory antibody responses following quadrivalent influenza vaccination but lower immunogenicity among individuals with lower CD4+ T-cell counts (15). Our study extends these findings by providing contemporary evidence on the immunogenicity and safety of IIV3 in Chinese adults living with HIV and demonstrating strain-specific differences in vaccine-induced immune responses compared with HIV-uninfected adults.

The most important finding of this study was the marked strain-specific variation in vaccine-induced immune responses. Although seroconversion against A(H1N1) was high in adults living with HIV and did not differ significantly between groups, antibody responses to A(H3N2) and B/Victoria were generally lower in adults living with HIV than in HIV-uninfected adults. Similar findings have been reported previously, particularly for A(H3N2) (5, 15). These strain-specific differences may reflect a combination of host-, virus-, and exposure-related factors rather than HIV-associated immune dysfunction alone. Several mechanisms may contribute to the observed differences. Chronic HIV infection is associated with persistent immune activation and impaired B-cell and T follicular helper cell function, which may compromise high-quality antibody production despite effective ART (16, 17). In addition, A(H3N2) viruses undergo frequent antigenic drift, resulting in substantial antigenic diversity and contributing to the lower and more variable vaccine effectiveness observed for A(H3N2) than for other influenza subtypes (18, 19). Vaccine strain composition may also be relevant. Compared with the 2023–2024 Northern Hemisphere season, the A(H3N2) component of egg-based vaccines was updated from an A/Darwin/9/2021-like virus to an A/Thailand/8/2022-like virus for the 2024–2025 season, whereas the A(H1N1)pdm09 and B/Victoria components remained unchanged (10, 20). The recent update of the A(H3N2) vaccine component indicates that differences in prior antigenic exposure and pre-existing immunity may be relevant when interpreting the strain-specific responses. However, because both groups received the same vaccine and HI responses were assessed against homologous vaccine strains, antigenic drift alone is unlikely to explain the between-group differences. The unchanged B/Victoria component further suggests that recent vaccine-strain updating cannot account for all of the observed strain-specific variation. Accordingly, the comparatively lower responses to A(H3N2) and B/Victoria likely reflect multiple contributing factors, and the relative contribution of HIV-associated immune dysfunction cannot be determined from the present study. These strain-specific findings should therefore be considered hypothesis-generating and require confirmation across multiple influenza seasons and independent cohorts.

The complementary global three-strain analysis provided an integrated assessment of humoral responses across the three vaccine strains. Although global response scores were lower in participants living with HIV at both Day 0 and Day 28, the Group × Time interaction was not statistically significant, indicating that the magnitude of increase from baseline did not differ significantly between groups in the primary global-response analysis. Notably, the lower global response observed in adults living with HIV at baseline persisted at Day 28, as confirmed by the model-based between-group contrast. In contrast, Tawfik et al. reported that the baseline difference between previously unvaccinated and vaccinated healthy adults was no longer significant by Day 90 (12), although direct comparison is limited by differences in study populations and assessment time points. Consistent with the global response findings, a substantially lower proportion of participants living with HIV achieved seroprotection against all three vaccine strains at Day 28. Together with the strain-specific analyses, the global three-strain assessment provides an integrated view of humoral immunity, showing that both groups mounted substantial antibody responses after vaccination, while between-group differences in the overall level and breadth of responses remained evident.

Beyond the descriptive comparisons, multivariable analyses demonstrated that HIV infection remained independently associated with reduced vaccine-induced immune responses after adjustment for demographic characteristics, comorbidity, and baseline HI antibody levels. This finding indicates that the reduced immunogenicity observed in adults living with HIV was not fully accounted for by the measured baseline characteristics or baseline HI antibody levels. These findings support continued efforts to better understand and optimize influenza vaccine responses in people living with HIV, particularly for strains showing comparatively lower immunogenicity.

In exploratory analyses restricted to participants living with HIV, recent CD4+ T-cell count, time since HIV diagnosis, and ART duration were not independently associated with vaccine-induced immune responses after adjustment for other covariates. Previous studies have reported inconsistent associations between these HIV-related factors and influenza vaccine immunogenicity, with lower CD4+ T-cell counts more consistently linked to reduced immune responses, whereas evidence regarding ART duration remains limited (5, 15). The absence of significant associations in our study may reflect the relatively preserved immune status and well-controlled HIV infection in this cohort, in which all participants were receiving ART and all participants with evaluable HIV RNA were virologically suppressed (<200 copies/mL). In contrast, baseline HI antibody level was consistently associated with post-vaccination immune responses, suggesting an important role of pre-existing influenza immunity in short-term vaccine responses. These exploratory findings should be interpreted cautiously and require confirmation in larger and more diverse cohorts.

Baseline HI antibody level was consistently associated with vaccine-induced antibody responses in both the overall and HIV-restricted analyses. Higher baseline antibody levels were associated with greater odds of achieving seroprotection and higher post-vaccination antibody titers but smaller antibody increases after vaccination. This pattern has been consistently reported in influenza vaccine studies and likely reflects a ceiling effect, whereby individuals with higher pre-existing immunity have less potential for further fold increases despite achieving higher absolute antibody titers (21, 22). These findings highlight the importance of accounting for baseline HI antibody levels when evaluating vaccine immunogenicity and support their inclusion as a continuous covariate in multivariable analyses.

Older age was associated with reduced immune responses to A(H3N2) but enhanced responses to B/Victoria. Although immunosenescence is known to influence influenza vaccine immunogenicity, age-related effects vary across influenza strains and study populations (23, 24). Although age was a prespecified stratification factor, the study was not specifically powered to detect age-related differences in vaccine-induced immune responses. Therefore, these findings should be interpreted cautiously.

Consistent with previous evidence, IIV3 was well tolerated in adults living with HIV, with no vaccine-related SAEs observed. These findings are consistent with systematic reviews demonstrating the favorable safety profile of inactivated influenza vaccines in this population and further support current recommendations for annual influenza vaccination (5, 6, 13, 14).

This study has several strengths. To our knowledge, it is one of the largest comparative studies evaluating the immunogenicity and safety of seasonal IIV3 in adults living with HIV in China. The inclusion of an age- and sex-comparable HIV-uninfected group recruited during the same period and from the same geographic area, together with adjustment for baseline HI antibody levels and other clinically relevant covariates, enabled a more rigorous assessment of the association between HIV infection and vaccine-induced immune responses than unadjusted comparisons alone.

Several limitations should also be acknowledged. First, this was a single-center study, which may limit the generalizability of the findings. Second, because the between-group comparison was non-randomized, residual confounding due to unmeasured or incompletely measured factors cannot be excluded despite adjustment for measured baseline characteristics. Several potentially relevant factors, including nadir CD4+ T-cell count, AIDS history, specific ART regimens, smoking status, nutritional status, body mass index, and prior influenza exposure, were not systematically collected. Although participants in both groups had not received influenza vaccination within the preceding year, earlier influenza vaccination history was not systematically collected and could not be accounted for in the analyses. Third, immunogenicity was assessed primarily by HI antibody responses at 28 days after vaccination, whereas cellular immunity and long-term antibody persistence were not evaluated. Fourth, the very small number of ILI events and the absence of virological confirmation precluded meaningful assessment of vaccine efficacy against influenza; therefore, the ILI findings should be considered descriptive only. The unvaccinated cohort of adults living with HIV should be interpreted within the vaccination policy context in China at the time of study conduct, when seasonal influenza vaccination was recommended but remained voluntary and outside the National Immunization Program (9). Finally, the exploratory analyses among participants living with HIV may have been underpowered to detect modest associations between HIV-related clinical characteristics and vaccine-induced immune responses.

Despite these limitations, our findings provide contemporary evidence supporting the immunogenicity and safety of seasonal IIV3 in adults living with HIV in China and further highlight important strain-specific differences in vaccine-induced immune responses. These results support current recommendations for annual influenza vaccination in people living with HIV and provide a basis for future multicenter studies evaluating optimized vaccination strategies and long-term clinical protection (6, 13).

## Conclusion

In conclusion, adults living with HIV developed substantial antibody responses following IIV3 vaccination, although the magnitude of vaccine-induced immune responses varied across influenza strains. Compared with HIV-uninfected adults, adults living with HIV showed high seroconversion against A(H1N1), whereas immune responses to A(H3N2) and B/Victoria were generally lower. HIV infection remained independently associated with reduced vaccine-induced immune responses for several strain-specific outcomes after adjustment for demographic and clinical factors, although residual confounding from unmeasured factors cannot be excluded. Baseline HI antibody level was consistently associated with post-vaccination antibody responses. IIV3 demonstrated a favorable safety profile and was well tolerated in adults living with HIV. These findings provide contemporary evidence supporting annual influenza vaccination in adults living with HIV in China and highlight the importance of considering strain-specific differences when evaluating vaccine immunogenicity in this population. Further multicenter studies with longer follow-up are warranted to assess the durability of immune responses and their relationship with clinical protection.

## Data Availability

The raw data supporting the conclusions of this article will be made available by the authors, without undue reservation.
