# Supplementary material for: Immunogenicity and safety of trivalent inactivated influenza vaccine in adults living with HIV compared with HIV-uninfected adults: a study from China
Source: Front Immunol. 2026 Oct 16;17:1945123. doi: 10.3389/fimmu.2026.1945123 (PMC13719463; doi:10.3389/fimmu.2026.1945123)
Supplement: Supplementary file 1 [file Supplementaryfile1.docx]

**Supplementary Table S1. Baseline comorbidities in vaccinated participants living with HIV and HIV-uninfected participants in the PPS.**

| **Comorbidity** | **Group 1 (n=200)** | **Group 2 (n=248)** | ***P* value** |
| --- | --- | --- | --- |
| Any comorbidity, n (%) | 52 (26.00) | 60 (24.19) | 0.663 |
| Hypertension, n (%) | 32 (16.00) | 41 (16.53) | 0.879 |
| Diabetes mellitus, n (%) | 9 (4.50) | 15 (6.05) | 0.469 |
| Dyslipidaemia, n (%) | 2 (1.00) | 10 (4.03) | 0.048 |
| Hyperuricaemia, n (%) | 1 (0.50) | 3 (1.21) | 0.632 |
| HBV infection, n (%) | 5 (2.50) | 1 (0.40) | 0.094 |
| HCV infection, n (%) | 0 (0.00) | 0 (0.00) | — |
| Other cardiovascular diseases, n (%) | 1 (0.50) | 1 (0.40) | — |
| Other endocrine and metabolic diseases, n (%) | 3 (1.50) | 1 (0.40) | — |
| Gastrointestinal diseases, n (%) | 0 (0.00) | 1 (0.40) | — |
| Musculoskeletal diseases, n (%) | 0 (0.00) | 2 (0.81) | — |
| Respiratory diseases, n (%) | 0 (0.00) | 1 (0.40) | — |
| Psychiatric and neurological diseases, n (%) | 1 (0.50) | 0 (0.00) | — |
| Neoplasms, n (%) | 1 (0.50) | 0 (0.00) | — |
| Genitourinary diseases, n (%) | 0 (0.00) | 2 (0.81) | — |
| Skin diseases, n (%) | 1 (0.50) | 0 (0.00) | — |
| Other infections, n (%) | 2 (1.00) | 0 (0.00) | — |

PPS, per-protocol set; HIV, human immunodeficiency virus; HBV, Hepatitis B Virus; HCV, Hepatitis C Virus.

Participants could have more than one comorbidity; therefore, comorbidity categories were not mutually exclusive and their counts may not sum to the number of participants with any comorbidity.

*P* values for common and clinically relevant comorbidities were calculated using the Pearson *χ²* test or Fisher's exact test, as appropriate. Rare comorbidity categories are presented descriptively because of the small number of participants with these conditions.
