# Supplementary material for: Immunogenicity and safety of trivalent inactivated influenza vaccine in adults living with HIV compared with HIV-uninfected adults: a study from China
Source: Front Immunol. 2026 Oct 16;17:1945123. doi: 10.3389/fimmu.2026.1945123 (PMC13719463; doi:10.3389/fimmu.2026.1945123)
Supplement: Supplementary file 2 [file Supplementaryfile2.docx]

**Supplementary Table S2. Immunogenicity stratified by baseline HI antibody titer at 28 days after vaccination in the PPS (<1:10 vs ≥1:10).**

| **Outcome** | **Group 1 (n=200)** | **Group 2 (n=248)** | ***P* value** |
| --- | --- | --- | --- |
| **Pre-vaccination HI titer <1:10** | | | |
| **Seroconversion rate, n/N (%) [95% CI]** | | | |
| A(H1N1) | 130/142 (91.55) [86.97–96.13] | 59/63 (93.65) [87.63–99.67] | 0.605 |
| A(H3N2) | 32/72 (44.44) [33.16–55.72] | 53/56 (94.64) [88.75–100.00] | <0.001 |
| B/Victoria | 69/188 (36.70) [29.81–43.59] | 132/208 (63.46) [56.92–69.99] | <0.001 |
| **Seroprotection rate, n/N (%) [95% CI]** | | | |
| A(H1N1) | 130/142 (91.55) [86.97–96.13] | 59/63 (93.65) [87.63–99.67] | 0.605 |
| A(H3N2) | 32/72 (44.44) [33.16–55.72] | 53/56 (94.64) [88.75–100.00] | <0.001 |
| B/Victoria | 69/188 (36.70) [29.81–43.59] | 132/208 (63.46) [56.92–69.99] | <0.001 |
| **GMT (95% CI)** | | | |
| A(H1N1) | 183.43 (145.91–230.61) | 268.35 (193.74–371.69) | 0.065 |
| A(H3N2) | 27.48 (17.51–43.13) | 195.04 (136.49–278.72) | <0.001 |
| B/Victoria | 22.84 (18.14–28.76) | 49.67 (40.43–61.04) | <0.001 |
| **GMI (95% CI)** | | | |
| A(H1N1) | 36.69 (29.18–46.12) | 53.67 (38.75–74.34) | 0.065 |
| A(H3N2) | 5.50 (3.50–8.63) | 39.01 (27.30–55.74) | <0.001 |
| B/Victoria | 4.57 (3.63–5.75) | 9.93 (8.09–12.21) | <0.001 |
| **Pre-vaccination HI titer ≥1:10** | | | |
| **Seroconversion rate, n/N (%) [95% CI]** | | | |
| A(H1N1) | 52/58 (89.66) [82.23–97.09] | 156/185 (84.32) [79.09–89.55] | 0.313 |
| A(H3N2) | 67/128 (52.34) [43.69–61.00] | 169/192 (88.02) [83.43–92.61] | <0.001 |
| B/Victoria | 11/12 (91.67) [76.03–100.00] | 28/40 (70.00) [55.80–84.20] | 0.129 |
| **Seroprotection rate, n/N (%) [95% CI]** | | | |
| A(H1N1) | 58/58 (100.00) [100.00–100.00] | 184/185 (99.46) [98.40–100.00] | 0.575 |
| A(H3N2) | 95/128 (74.22) [66.64–81.80] | 188/192 (97.92) [95.90–99.94] | <0.001 |
| B/Victoria | 12/12 (100.00) [100.00–100.00] | 38/40 (95.00) [88.25–100.00] | 0.430 |
| **GMT (95% CI)** | | | |
| A(H1N1) | 469.07 (328.88–669.02) | 518.87 (442.37–608.59) | 0.565 |
| A(H3N2) | 107.76 (79.62–145.84) | 386.08 (325.58–457.83) | <0.001 |
| B/Victoria | 179.59 (81.26–396.92) | 162.80 (115.19–230.08) | 0.791 |
| **GMI (95% CI)** | | | |
| A(H1N1) | 23.74 (16.15–34.88) | 13.98 (11.28–17.33) | 0.018 |
| A(H3N2) | 3.49 (2.70–4.51) | 10.23 (8.69–12.03) | <0.001 |
| B/Victoria | 11.31 (4.15–30.83) | 7.46 (4.84–11.52) | 0.374 |

HI, hemagglutination inhibition; PPS, per-protocol set; CI, confidence interval; GMT, geometric mean titer; GMI, geometric mean increase.

Subgroup analyses should be interpreted with caution because of the limited sample size in some strata. For participants with baseline HI titers <1:10, seroconversion and seroprotection rates may be identical because seroconversion was defined as achieving a post-vaccination HI titer ≥1:40.
