# Supplementary material for: Immunogenicity and safety of trivalent inactivated influenza vaccine in adults living with HIV compared with HIV-uninfected adults: a study from China
Source: Front Immunol. 2026 Oct 16;17:1945123. doi: 10.3389/fimmu.2026.1945123 (PMC13719463; doi:10.3389/fimmu.2026.1945123)
Supplement: Supplementary file 3 [file Supplementaryfile3.docx]

**Supplementary Table S3. Immunogenicity stratified by baseline HI antibody titer at 28 days after vaccination in the PPS (<1:40 vs ≥1:40).**

| **Outcome** | **Group 1 (n=200)** | **Group 2 (n=248)** | ***P* value** |
| --- | --- | --- | --- |
| **Pre-vaccination HI titer <1:40** | | | |
| **Seroconversion rate, n/N (%) [95% CI]** | | | |
| A(H1N1) | 171/185 (92.43) [88.62–96.24] | 154/161 (95.65) [92.50–98.80] | 0.211 |
| A(H3N2) | 65/143 (45.45) [37.29–53.62] | 123/131 (93.89) [89.79–98.00] | <0.001 |
| B/Victoria | 79/198 (39.90) [33.08–46.72] | 154/235 (65.53) [59.46–71.61] | <0.001 |
| **Seroprotection rate, n/N (%) [95% CI]** | | | |
| A(H1N1) | 173/185 (93.51) [89.96–97.06] | 156/161 (96.89) [94.21–99.57] | 0.147 |
| A(H3N2) | 72/143 (50.35) [42.15–58.55] | 125/131 (95.42) [91.84–99.00] | <0.001 |
| B/Victoria | 79/198 (39.90) [33.08–46.72] | 157/235 (66.81) [60.79–72.83] | <0.001 |
| **GMT (95% CI)** | | | |
| A(H1N1) | 221.66 (180.64, 271.99) | 383.42 (315.12, 466.53) | <0.001 |
| A(H3N2) | 37.38 (27.52, 50.75) | 214.05 (171.20, 267.62) | <0.001 |
| B/Victoria | 25.38 (20.13, 31.99) | 55.82 (46.02, 67.72) | <0.001 |
| **GMI (95% CI)** | | | |
| A(H1N1) | 35.14 (28.80–42.88) | 37.69 (30.83–46.08) | 0.627 |
| A(H3N2) | 4.30 (3.19–5.80) | 22.21 (17.57–28.07) | <0.001 |
| B/Victoria | 4.87 (3.88–6.10) | 10.13 (8.38–12.25) | <0.001 |
| **Pre-vaccination HI titer ≥1:40** | | | |
| **Seroconversion rate, n/N (%) [95% CI]** | | | |
| A(H1N1) | 11/15 (73.33) [50.95–95.71] | 61/87 (70.11) [60.50–79.72] | 0.801 |
| A(H3N2) | 34/57 (59.65) [46.91–72.39] | 99/117 (84.62) [78.08–91.15] | <0.001 |
| B/Victoria | 1/2 (50.00) [0.00–100.00] | 6/13 (46.15) [19.05–73.25] | 0.919 |
| **Seroprotection rate, n/N (%) [95% CI]** | | | |
| A(H1N1) | 15/15 (100.00) [100.00–100.00] | 87/87 (100.00) [100.00–100.00] | NE |
| A(H3N2) | 55/57 (96.49) [92.71–100.00] | 116/117 (99.15) [97.48–100.00] | 0.207 |
| B/Victoria | 2/2 (100.00) [100.00–100.00] | 13/13 (100.00) [100.00–100.00] | NE |
| **GMT (95% CI)** | | | |
| A(H1N1) | 670.27 (317.76–1413.84) | 563.40 (456.33–695.59) | 0.554 |
| A(H3N2) | 273.21 (199.05–375.00) | 538.97 (446.71–650.28) | <0.001 |
| B/Victoria | 160.00 (NE) | 232.39 (136.75–394.91) | 0.570 |
| **GMI (95% CI)** | | | |
| A(H1N1) | 11.58 (4.86–27.61) | 5.91 (4.54–7.70) | 0.066 |
| A(H3N2) | 3.67 (2.69–5.01) | 8.14 (6.74–9.84) | <0.001 |
| B/Victoria | 2.00 (0.00–13365.30) | 2.90 (1.51–5.59) | 0.655 |

HI, hemagglutination inhibition; PPS, per-protocol set; CI, confidence interval; NE, not estimable; GMT, geometric mean titer; GMI, geometric mean increase.

Subgroup analyses should be interpreted with caution because of the limited sample size in some strata. Confidence intervals could not be estimated for some subgroups because of the very small sample size. NE, not estimable because confidence intervals could not be calculated due to the limited sample size.
