# Supplementary material for: Immunogenicity and safety of trivalent inactivated influenza vaccine in adults living with HIV compared with HIV-uninfected adults: a study from China
Source: Front Immunol. 2026 Oct 16;17:1945123. doi: 10.3389/fimmu.2026.1945123 (PMC13719463; doi:10.3389/fimmu.2026.1945123)
Supplement: Supplementary file 4 [file Supplementaryfile4.docx]

**Supplementary Table S4. Global three-strain HI antibody responses following IIV3 vaccination in participants living with HIV and HIV-uninfected participants in the PPS.**

| **Outcome** | **Time point/effect** | **Group 1 (n=200)** | **Group 2 (n=248)** | **Estimate (95% CI)** | ***P* value** |
| --- | --- | --- | --- | --- | --- |
| Global three-strain response score, mean ± SD | | | | | |
|  | Day 0 | 1.14 ± 1.41 | 2.39 ± 1.35 | — | — |
|  | Day 28 | 4.77 ± 1.45 | 6.27 ± 1.50 | — | — |
|  | Change from Day 0 to Day 28 | 3.62 ± 1.54 | 3.88 ± 1.74 | — | — |
| Mixed-effects model (fixed effects) | | | | | |
|  | Group effect | — | — | −1.249 (−1.515, −0.983) | <0.001 |
|  | Time effect | — | — | 3.879 (3.674, 4.084) | <0.001 |
|  | Group × Time interaction | — | — | −0.256 (−0.563, 0.052) | 0.103 |
| Model-based between-group contrast | | | | | |
|  | Group 1 vs. Group 2 at Day 28 | — | — | −1.505 (−1.770, −1.239) | <0.001 |

HI, hemagglutination inhibition; IIV3, trivalent inactivated influenza vaccine; ORQ, ordered quantile; PPS, per-protocol set; CI, confidence interval; SD, standard deviation.

Data are presented as mean ± SD unless otherwise indicated. The global three-strain response score was constructed using an approach adapted from Tawfik et al. ^[12]^. HI antibody titers for A(H1N1), A(H3N2), and B/Victoria were transformed into ordinal scores ranging from 0 (HI titer <1:10) to 10 (HI titer 1:5120), with one-unit increments corresponding to two-fold increases in HI titer. The mean ordinal score across the three strains was combined with a seroprotection weight of 1 if at least one strain achieved an HI titer ≥1:40, or 0 otherwise, followed by ordered quantile (ORQ) normalization.

The linear mixed-effects model included Group, Time, and Group × Time as fixed effects and participant as a random intercept, with Group 2 and Day 0 as the reference levels. The Group effect represents the between-group difference at Day 0, whereas the Time effect represents the change from Day 0 to Day 28 in Group 2. The Group × Time interaction represents the difference in changes from baseline between the two groups. The Day 28 between-group contrast was estimated from the same model as the sum of the Group and Group × Time coefficients and represents the difference in estimated global three-strain response scores between Group 1 and Group 2 (Group 1 − Group 2). All 95% CIs were calculated using normal-distribution critical values. Model estimates are expressed as mean differences in global response scores.
