# Supplementary material for: Immunogenicity and safety of trivalent inactivated influenza vaccine in adults living with HIV compared with HIV-uninfected adults: a study from China
Source: Front Immunol. 2026 Oct 16;17:1945123. doi: 10.3389/fimmu.2026.1945123 (PMC13719463; doi:10.3389/fimmu.2026.1945123)
Supplement: Supplementary file 5 [file Supplementaryfile5.docx]

**Supplementary Table S5. Distribution of the number of vaccine strains achieving seroprotection at Day 28 in participants living with HIV and HIV-uninfected participants in the PPS.**

| **Number of strains achieving seroprotection at Day 28** | **Group 1 (n=200)** | **Group 2 (n=248)** | ***P* value** |
| --- | --- | --- | --- |
| 0, n (%) | 3 (1.50) | 0 (0.00) |  |
| 1, n (%) | 42 (21.00) | 6 (2.42) |  |
| 2, n (%) | 111 (55.50) | 78 (31.45) |  |
| 3, n (%) | 44 (22.00) | 164 (66.13) |  |
| Overall distribution | — | — | <0.001 |
| Seroprotection against all three strains, n (%) | 44 (22.00) | 164 (66.13) | <0.001 |

HI, hemagglutination inhibition; PPS, per-protocol set.

Seroprotection was defined as an HI antibody titer ≥1:40 for the corresponding vaccine strain at Day 28. The number of strains achieving seroprotection represents the number of vaccine strains [A(H1N1), A(H3N2), and B/Victoria] for which each participant achieved an HI titer ≥1:40 at Day 28. The overall distribution of participants achieving seroprotection against 0, 1, 2, or 3 strains was compared between groups using the Pearson chi-square test. The proportion of participants achieving seroprotection against all three strains was compared between groups using the Pearson chi-square test with Yates’ continuity correction.
