# Supplementary material for: Immunogenicity and safety of trivalent inactivated influenza vaccine in adults living with HIV compared with HIV-uninfected adults: a study from China
Source: Front Immunol. 2026 Oct 16;17:1945123. doi: 10.3389/fimmu.2026.1945123 (PMC13719463; doi:10.3389/fimmu.2026.1945123)
Supplement: Supplementary file 6 [file Supplementaryfile6.docx]

**Supplementary Table S6. HIV-related clinical characteristics of vaccinated participants living with HIV in the PPS.**

| **Characteristic** | **Group 1 (n=200)** |
| --- | --- |
| Time since HIV diagnosis, years, median (IQR) | 8.66 (4.49–11.95) |
| Receiving ART, n (%) | 200 (100.00) |
| ART duration, years, median (IQR) | 8.48 (4.36–10.52) |
| Recent CD4+ T-cell count, cells/μL, median (IQR) | 557.0 (394.0–702.0) |
| CD4+ T-cell count <500 cells/μL, n (%) | 79 (39.50) |
| CD4+ T-cell count ≥500 cells/μL, n (%) | 114 (57.00) |
| HIV RNA result available, n/N (%) | 199/200 (99.50) |
| HIV RNA undetectable, n/N (%) | 189/199 (94.97) |
| Virologically suppressed (<200 copies/mL), n/N (%) | 199/199 (100.00) |
| HBV coinfection, n (%) | 5 (2.50) |
| HCV coinfection, n (%) | 0 (0.00) |
| Any comorbidity, n (%) | 52 (26.00) |
| Any concomitant medication, n (%) | 50 (25.00) |

PPS, per-protocol set; ART, antiretroviral therapy; HIV, human immunodeficiency virus; HBV, hepatitis B virus; HCV, hepatitis C virus; IQR, interquartile range.

Time since HIV diagnosis and ART duration were evaluable for 158 participants. Recent CD4+ T-cell count was available for 193 participants. HIV RNA results were available for 199 participants. The denominator for HIV RNA undetectability and virological suppression was the number of participants with evaluable HIV RNA results (n=199). Virological suppression was defined as HIV RNA <200 copies/mL. Concomitant medication use was summarized based on available concomitant medication records; medication start dates were incomplete for some records.
