# Supplementary material for: Immunogenicity and safety of trivalent inactivated influenza vaccine in adults living with HIV compared with HIV-uninfected adults: a study from China
Source: Front Immunol. 2026 Oct 16;17:1945123. doi: 10.3389/fimmu.2026.1945123 (PMC13719463; doi:10.3389/fimmu.2026.1945123)
Supplement: Supplementary file 7 [file Supplementaryfile7.docx]

**Supplementary Table S7. Multivariable analyses of factors associated with vaccine-induced HI antibody responses among IIV3 recipients living with HIV in the PPS.**

| **Variable** | **A(H1N1)** | | **A(H3N2)** | | **B/Victoria** | |
| --- | --- | --- | --- | --- | --- | --- |
|  | **Estimate (95% CI)** | ***P* value** | **Estimate (95% CI)** | ***P* value** | **Estimate (95% CI)** | ***P* value** |
| **Seroconversion, aOR (95% CI)** |  |  |  |  |  |  |
| Age ≥50 years | 8.93 (1.09–72.94) | 0.041 | 0.24 (0.12–0.51) | <0.001 | 2.61 (1.26–5.38) | 0.009 |
| Male sex | NE | — | 0.51 (0.03–8.98) | 0.645 | 1.01 (0.06–17.99) | 0.992 |
| Comorbidity | 1.12 (0.21–5.92) | 0.894 | 0.97 (0.40–2.31) | 0.937 | 0.91 (0.38–2.18) | 0.836 |
| Baseline log_2_HI titer | 0.62 (0.39–0.97) | 0.035 | 1.02 (0.83–1.24) | 0.882 | 1.48 (0.74–2.99) | 0.272 |
| Baseline CD4+ T-cell count <500 cells/μL | 0.58 (0.18–1.87) | 0.358 | 0.64 (0.32–1.30) | 0.220 | 1.46 (0.72–2.93) | 0.294 |
| Time since HIV diagnosis <5 years | 0.63 (0.15–2.57) | 0.515 | 1.62 (0.62–4.21) | 0.325 | 1.08 (0.42–2.79) | 0.873 |
| ART duration <2 years | 1.58 (0.23–10.83) | 0.639 | 0.75 (0.20–2.88) | 0.679 | 2.77 (0.75–10.27) | 0.127 |
| **Seroprotection, aOR (95% CI)** |  |  |  |  |  |  |
| Age ≥50 years | 4.32 (0.50–37.74) | 0.186 | 0.13 (0.05–0.33) | <0.001 | 2.32 (1.09–4.94) | 0.029 |
| Male sex | NE | — | 1.58 (0.03–84.15) | 0.821 | 0.78 (0.04–13.82) | 0.865 |
| Comorbidity | 1.82 (0.19–17.60) | 0.604 | 0.69 (0.24–1.97) | 0.490 | 0.90 (0.37–2.20) | 0.811 |
| Baseline log_2_HI titer | NE | — | 2.56 (1.72–3.83) | <0.001 | NE | — |
| Baseline CD4+ T-cell count <500 cells/μL | 0.34 (0.08–1.51) | 0.156 | 0.42 (0.18–1.01) | 0.053 | 1.34 (0.65–2.77) | 0.428 |
| Time since HIV diagnosis <5 years | 0.29 (0.06–1.57) | 0.152 | 1.75 (0.49–6.22) | 0.390 | 0.78 (0.27–2.25) | 0.644 |
| ART duration <2 years | 1.58 (0.12–21.32) | 0.732 | 0.43 (0.07–2.63) | 0.362 | 3.68 (0.91–14.86) | 0.068 |
| **GMT, β (95% CI)** |  |  |  |  |  |  |
| Age ≥50 years | 0.39 (−0.29–1.06) | 0.263 | −2.15 (−2.91–−1.39) | <0.001 | 0.86 (0.02–1.69) | 0.045 |
| Male sex | −0.23 (−3.13–2.68) | 0.878 | −0.58 (−3.75–2.60) | 0.720 | 0.69 (−2.82–4.20) | 0.698 |
| Comorbidity | −0.75 (−1.55–0.06) | 0.071 | 0.44 (−0.46–1.34) | 0.332 | −0.01 (−1.00–0.99) | 0.993 |
| Baseline log_2_HI titer | 0.49 (0.19–0.78) | 0.001 | 0.74 (0.53–0.96) | <0.001 | 1.08 (0.30–1.86) | 0.007 |
| Baseline CD4+ T-cell count <500 cells/μL | −0.39 (−1.05–0.27) | 0.240 | −0.33 (−1.07–0.41) | 0.377 | 0.38 (−0.43–1.19) | 0.358 |
| Time since HIV diagnosis <5 years | −0.51 (−1.39–0.36) | 0.248 | 0.73 (−0.24–1.70) | 0.140 | −0.05 (−1.12–1.02) | 0.923 |
| ART duration <2 years | 0.00 (−1.24–1.24) | 0.998 | −0.54 (−1.91–0.83) | 0.439 | 0.70 (−0.82–2.21) | 0.366 |
| **GMI, β (95% CI)** |  |  |  |  |  |  |
| Age ≥50 years | 0.39 (−0.29–1.06) | 0.263 | −2.15 (−2.91–−1.39) | <0.001 | 0.86 (0.02–1.69) | 0.045 |
| Male sex | −0.23 (−3.13–2.68) | 0.878 | −0.58 (−3.75–2.60) | 0.720 | 0.69 (−2.82–4.20) | 0.698 |
| Comorbidity | −0.75 (−1.55–0.06) | 0.071 | 0.44 (−0.46–1.34) | 0.332 | −0.01 (−1.00–0.99) | 0.993 |
| Baseline log_2_HI titer | −0.51 (−0.81–−0.22) | <0.001 | −0.26 (−0.47–−0.05) | 0.018 | 0.08 (−0.70–0.86) | 0.842 |
| Baseline CD4+ T-cell count <500 cells/μL | −0.39 (−1.05–0.27) | 0.240 | −0.33 (−1.07–0.41) | 0.377 | 0.38 (−0.43–1.19) | 0.358 |
| Time since HIV diagnosis <5 years | −0.51 (−1.39–0.36) | 0.248 | 0.73 (−0.24–1.70) | 0.140 | −0.05 (−1.12–1.02) | 0.923 |
| ART duration <2 years | 0.00 (−1.24–1.24) | 0.998 | −0.54 (−1.91–0.83) | 0.439 | 0.70 (−0.82–2.21) | 0.366 |

HI, hemagglutination inhibition; GMT, geometric mean titer; GMI, geometric mean increase; PPS, per-protocol set; aOR, adjusted odds ratio; β, regression coefficient; CI, confidence interval.

Models included age, sex, comorbidity, baseline log_2_ HI antibody titer, baseline CD4+ T-cell count, time since HIV diagnosis, and ART duration. Reference categories were age 18–49 years, female sex, no comorbidity, baseline CD4+ T-cell count ≥500 cells/μL, time since HIV diagnosis ≥5 years, and ART duration ≥2 years. Baseline log_2_ HI antibody titer was modeled as a continuous variable. Parameters labeled as NE were not estimable because of sparse data or quasi-complete separation in the logistic regression models.
