# Supplementary material for: Immunogenicity and safety of trivalent inactivated influenza vaccine in adults living with HIV compared with HIV-uninfected adults: a study from China
Source: Front Immunol. 2026 Oct 16;17:1945123. doi: 10.3389/fimmu.2026.1945123 (PMC13719463; doi:10.3389/fimmu.2026.1945123)
Supplement: Supplementary file 8 [file Supplementaryfile8.docx]

**Supplementary Table S8. Severity of vaccine-related adverse reactions within 7 days after vaccination in the SS.**

| **AEs** | **Group 1 (n=237)** | | |  | **Group 2 (n=251)** | | |
| --- | --- | --- | --- | --- | --- | --- | --- |
|  | **Grade 1** | **Grade 2** | **Grade ≥3** |  | **Grade 1** | **Grade 2** | **Grade ≥3** |
| **Local, n (%)** |  |  |  |  |  |  |  |
| injection-site pain | 11 (4.64%) | 0 (0.00%) | 1 (0.42%) |  | 15 (5.98%) | 0 (0.00%) | 0 (0.00%) |
| induration | 5 (2.11%) | 1 (0.42%) | 0 (0.00%) |  | 3 (1.20%) | 0 (0.00%) | 0 (0.00%) |
| swelling | 5 (2.11%) | 1 (0.42%) | 0 (0.00%) |  | 3 (1.20%) | 0 (0.00%) | 0 (0.00%) |
| rash | 2 (0.84%) | 0 (0.00%) | 0 (0.00%) |  | 0 (0.00%) | 0 (0.00%) | 0 (0.00%) |
| erythema | 4 (1.69%) | 0 (0.00%) | 0 (0.00%) |  | 1 (0.40%) | 0 (0.00%) | 0 (0.00%) |
| pruritus | 3 (1.27%) | 0 (0.00%) | 0 (0.00%) |  | 0 (0.00%) | 0 (0.00%) | 0 (0.00%) |
| **Systemic, n (%)** |  |  |  |  |  |  |  |
| fever | 2 (0.84%) | 0 (0.00%) | 0 (0.00%) |  | 0 (0.00%) | 0 (0.00%) | 0 (0.00%) |
| cough | 6 (2.53%) | 2 (0.84%) | 0 (0.00%) |  | 1 (0.40%) | 0 (0.00%) | 0 (0.00%) |
| headache | 2 (0.84%) | 0 (0.00%) | 0 (0.00%) |  | 0 (0.00%) | 1 (0.40%) | 0 (0.00%) |
| myalgia | 6 (2.53%) | 0 (0.00%) | 1 (0.42%) |  | 1 (0.40%) | 0 (0.00%) | 0 (0.00%) |
| arthralgia | 1 (0.42%) | 0 (0.00%) | 0 (0.00%) |  | 1 (0.40%) | 0 (0.00%) | 0 (0.00%) |
| fatigue | 6 (2.53%) | 0 (0.00%) | 1 (0.42%) |  | 0 (0.00%) | 0 (0.00%) | 0 (0.00%) |
| nausea | 0 (0.00%) | 1 (0.42%) | 0 (0.00%) |  | 0 (0.00%) | 0 (0.00%) | 0 (0.00%) |
| vomiting | 0 (0.00%) | 0 (0.00%) | 1 (0.42%) |  | 0 (0.00%) | 0 (0.00%) | 0 (0.00%) |
| acute allergic reactions | 1 (0.42%) | 0 (0.00%) | 0 (0.00%) |  | 0 (0.00%) | 0 (0.00%) | 0 (0.00%) |
| **Unsolicited adverse events, n (%)** | 9 (3.80%) | 4 (1.69%) | 1 (0.42%) |  | 2 (0.80%) | 1 (0.40%) | 0 (0.00%) |

SS, safety set; AE, adverse event.

Data are presented as n (%), where n represents the number of participants experiencing the specified adverse reaction. Participants may have experienced more than one adverse reaction. Grade ≥3 includes all adverse reactions of Grade 3 or higher severity. No Grade 4 vaccine-related adverse reactions were reported in either group.
