# Supplementary material for: Immunogenicity and safety of trivalent inactivated influenza vaccine in adults living with HIV compared with HIV-uninfected adults: a study from China
Source: Front Immunol. 2026 Oct 16;17:1945123. doi: 10.3389/fimmu.2026.1945123 (PMC13719463; doi:10.3389/fimmu.2026.1945123)
Supplement: Supplementary file 9 [file Supplementaryfile9.docx]

**Supplementary Table S9. Descriptive summary of influenza-like illness during the period from 29 days to 6 months after vaccination among vaccinated and unvaccinated participants living with HIV in the PPS.**

| **Outcome** | **Vaccinated participants living with HIV (n=200 )** | **Unvaccinated participants living with HIV (n=246 )** |
| --- | --- | --- |
| ILI, n (%) | 1 (0.50) | 2 (0.81) |

ILI, influenza-like illness; PPS, per-protocol set.

ILI was defined as fever (≥38.0°C) accompanied by cough or sore throat. ILI was identified based on clinical symptoms without virological confirmation. Data are presented descriptively as exploratory, hypothesis-generating observations; no formal hypothesis testing was performed.
